# Speech language pathologists’ attitudes on bilingual practices for children with autism in India: A qualitative study

**DOI:** 10.1371/journal.pone.0343695

**Published:** 2026-03-26

**Authors:** Kaluva Bindu Sree, Sebastian Padickaparambil, Shivani Tiwari

**Affiliations:** 1 Department of Speech and Hearing, Manipal College of Health Professions, Manipal Academy of Higher Education (MAHE), Manipal, Karnataka, India; 2 Department of Clinical Psychology, Manipal College of Health Professions, Manipal Academy of Higher Education (MAHE), Manipal, Karnataka, India; Father Muller Charitable Institutions, INDIA

## Abstract

The present study, aimed to investigate SLPs’ attitude regarding bilingual practices in children with autism. We conducted in-depth telephonic interviews on 13 SLPs working with autistic children in India having at least 2 years of work experience. The data obtained were subjected to stepwise inductive thematic analysis. Results of the study displayed five themes, viz. SLP’s opinion of monolingual vs. bilingual approaches in children with autism, Bilingual Practices for children with autism, Factors explaining the choice of language, Suggestions for parents of children with autism and future SLPs, and Need and scope for bilingual practice. Results of the study indicated SLP’s divergent attitude towards bilingual practices in children with autism, factors explaining the choice of language, such as lack of culturally appropriate assessment tools, and limited strategies for addressing parental concerns. These findings have implications to address awareness of evidence based bilingual practices in SLPs for children with ASD in multilingual settings, development of practice guidelines and standardized tests in underdeveloped languages. Furthermore, there is a need to educate and address concerns of parents and families of autistic children regarding bilingual approach.

## Introduction

India of one among the most linguistically diverse countries of the world [1], with over 1600 languages and dialects spoken by its people across 28 states and 8 union territories. The country hosts people with different religions and unique cultural identities represented through the array of languages spoken across its regions. Nearly 26% of India’s total population are bilingual speakers [2], and use of English is very common in the country. Bilingualism is the speaker’s ability to communicate in two languages [3]. Bilingualism is common and growing worldwide, with one in every three people being bilingual or multilingual [4]. Every child is exposed to a bilingual/multilingual environment in India [5].

Being bilingual is the norm, especially among young children [6]. Early exposure to two languages has been demonstrated to enhance a child’s mental and cognitive development. Parents of typically developing children show a favourable attitude towards bilingual exposure, stating the advantages of better possibilities for employment, better interactions with peers, and increased academic performance [7,8]. Bi/multilingualism is regarded as crucial for education and employment prospects in the complex sociocultural context of India [9]. It also fosters positive peer relationships, shapes one’s thinking, boosts confidence, and develops one’s reasoning ability, all of which help a child’s overall mental development [10]. Research demonstrates that early exposure to two or more languages does not confuse infants but promotes excellent language acquisition [11,12]. Furthermore, bi/multilinguals have an advantage over monolinguals in terms of greater literacy, academic success, social adaptability, and executive functioning [10,13].

The literature on bilingual children’s evidence-based practice with communication disorders presents mixed findings. According to Goldstein [14], a bilingual child will need more opportunities to be exposed to each language, resulting in two underdeveloped language systems. This leads to the belief that restricting input to a single language will provide more opportunities in that language, resulting in the development of at least one language system rather than none. Most parents of children with communication issues typically voice reservations about raising bilingual children. A few of the prevalent parental beliefs are that their child’s bilingualism or multilingualism results in a linguistic delay and further confusion that impairs their development [15]. Besides, many parents of children with communication disorders think their child’s linguistic development is hampered by bi/multilingualism since it confuses them [16].

An SLP is a professional who specializes in identification, evaluation and rehabilitation of individuals with communication disorders. SLP must be able to understand and incorporate the client’s preferred mode of communication. The SLP must also have the linguistic proficiency to distinguish between communication disorders and communication differences by selecting, administering and interpreting formal and informal assessment procedures correctly to describe everyday speech and language acquisition and the processes involved in oral and written language for both monolingual and bilingual speakers. The SLP should apply different intervention strategies by keeping in mind which language or mode of communication is most suitable and appropriate for the individual’s needs in the treatment of communication disorders [17].

Autism spectrum disorder (ASD) or autism is a neurodevelopmental disorder characterized by challenges in social communication and restricted, repetitive patterns of behaviour [18]. Children with autism can learn, think, and problem-solve in various ways, ranging from highly skilled to severely impaired [19,20]. The prevalence of autism in India in reported as 1 in 89 among children between 2–9 years of age [21]. Given the growing bilingual population and increased prevalence of autism, the role of healthcare professionals, such as SLP, is of relevance.

Although limited, research does not indicate a significant negative effect of bilingual exposure on language development for children with autism [22–25]. Despite the available information through evidence-based research, SLPs, often advice for a monolingual environment to the parents of children with autism, which could lead to practical issues and stress within families as well as negative impact on children with ASD [26,27]. Such practices are due to their belief that bilingualism is detrimental to the child, even though bilingualism has shown benefits [23]. SLP practices further show differences across cultural backgrounds. SLPs often lack an understanding of assessing children with bi/multilingual backgrounds due to constraints such as not knowing the language(s) spoken by the client, being unaware of practices involved in assessment and intervention, and paucity of available tools and resource materials [28,29]. SLPs often face challenges in making decisions regarding language choice consistently [27]. SLPs face challenges in assessing bilingual children and usually use standardized tests designed for something other than this population [30]. One of the reasons for using standardized tests in a different language were the need for more availability of local assessment tools and the lack of practice guidelines on the assessment of bilingual individuals [28,31]. Hence, research focusing on the knowledge, beliefs, and methods of SLPs in working with bilingual children with autism is gaining importance. Recently, the importance of professional development for early childhood health and education professionals in bilingualism and language development was emphasized [32].

There is only limited evidence to inform the best practices of SLPs for bilingual children with autism [33]. Furthermore, available findings from essentially monolingual contexts cannot be generalized to countries with divergent linguistic and cultural landscape. Given the multilingual and multicultural status of India, there is a need to understand SLPs’ knowledge, attitude and practices regarding bilingualism in children with autism to inform clinical practice. To address this gap the present study adopts a sociocultural framework to investigate SLPs’ attitudes and practices in bilingual children with autism. We selected a qualitative (thematic) analytical approach to capture the responses from the practising SLPs in India. The study specifically addressed the following research questions,

1) What are SLPs’ attitude, and practices regarding assessment and intervention of bilingual children with autism?
2) What are the factors influencing SLPs’ choice of language for assessment and intervention in children with autism having bilingual exposure?

## Method

This study employed a qualitative design where data was collected using in-depth interviews and was subjected to inductive thematic analysis. The Institutional Research Committee and the Institutional Ethics Committee reviewed and approved the study protocol (Kasturba Medical College and Kasturba Hospital, Institutional Ethics Committee 2; IEC no: 231/2022). Verbal consent(s) were obtained from each participant along with consent to audio record the interviews using google forms. The study was carried out from October 2022 to April 2023.

### Participants

Participants of this study were the practising SLPs working for bilingual children with autism. Participants for this study were recruited from all over India, working in different work setups, including private or institutional clinics, and Non-Governmental Organizations. SLPs were recruited by contacting alumni contacts, professional organization, i.e., Indian Speech and Hearing Association, and personal contacts. The selection criteria for inclusion in the study involved qualified SLPs with master’s degrees and at least 2 years of work experience with autistic children having bi/multilingual exposure. SLPs who have had worked with at least 10 children with ASD were recruited. SLPs were required to be bi/multilingual having good proficiency in English. A purposive sampling method was used to select SLPs based on the inclusion criteria.

### Procedure

A semi-structured interview guide was prepared initially along with follow-up probes based on the literature search and discussion within the research team. The draft of interview guide was validated by three expert SLPs having at least 5 years of clinical experience. Based on the expert response, the interview guide was modified, where the final version of the interview guide targeted SLPs’ attitude and practices regarding the assessment and intervention of children with autism having bilingual exposure (S1 Appendix). Primary researcher (a multilingual speech pathology graduate) conducted the interviews who had experience of working with autistic children having bi/multilingual exposure. The primary researcher completed a coursework on qualitative research methods, attended two workshops on qualitative analysis to understand the steps of coding and thematic analysis. To enhance familiarity with the interview methodology, the researcher conducted two mock (pilot) interviews on qualified SLPs who worked with autistic children having bilingual exposure. Eligible SLPs were invited to participate in the study through email communication. A Google form was emailed to all SLPs for collecting their background information (such as years of experience, work setup, place of work, etc.) as part of the inclusion criteria for the study. Verbal informed consent(s) were obtained from all SLPs over the telephone. In addition to verbal consent obtained over the telephone, explicit consent for recording the interviews was collected through the google sheets from the SLPs initially completed to provide their demographic details. After receiving the consent, primary researcher conducted one-to-one in-depth telephonic interviews at a time chosen by each SLP to ensure their convenience. The interview session started by explaining the purpose of the study and assuring the confidentiality of the data by the interviewer. All interviews were conducted over the telephone using the in-depth interview guide. The questioning strategy was responsive to each SLP, and primary researcher used follow-up questions to clarify comments and to elicit in-depth explanations as required. Respective probes were used based on how the SLP answered a question. An android application Cube ACR was used to record the oral consent and the telephonic interview(s). The average duration for each interview was approximately 30 minutes. All interviews were carried out in English. All data (audio files) were anonymised and stored in the laptop in a folder that was password protected and shared only within the research team.

### Coding and analysis

The primary investigator played back all recorded samples and manually transliterated them offline to ensure the accuracy of information. To ensure data integrity, an external reviewer was involved in the verification process. The transcriptions of the interviews were meticulously cross checked, and necessary corrections were provided to enhance the quality and accuracy of the transcribed data. The thematic analysis was conducted manually without the assistance of any external software or applications. The coding process was performed manually using excel sheets. Saturation of the data was observed after the tenth data. However, data were collected for additional 3 participants to ascertain the same. The data was analysed using a six-phase thematic analysis [34]. The transcripts were read systematically, and an initial set of codes were identified through line-by-line coding using an inductive thematic approach. An open coding process was followed. The meaning of each statement of the SLPs was determined based on the context and questions asked. Descriptive names/codes were assigned to each statement. Statements that illustrated the essence of each code were highlighted to locate the codes within transcripts. Two investigators independently coded the data, where codes from the two investigators showed above 80% agreement, while rest of the discrepancies were discussed among the research team. A code list was generated after coding all the data. Initial themes were derived from the codes. After the codes were obtained, these were discussed and reviewed based on the identified data. Codes were discussed within the research team, and all related codes were brought in and grouped to form sub-themes and themes explaining the research questions of the study (S2 Appendix). In the final presentation of the results, participants were anonymized, and excerpts were identified by the notation of P (participant SLP) with the corresponding number to ensure anonymity.

## Results

A total of 47 SLPs were approached to participate in the study, of which 16 SLPs provided their consent, and 15 of the consented SLPs participated. Thirteen of fifteen samples were included in the analysis; two could not be included due to poor audio quality and recording. Table 1 presents the demographic details of the SLP participants in the study. All SLPs had completed graduate degree in speech language pathology from India and were working in either private or institutional clinical set up. One of the SLPs worked in an NGO. None of the participants had undertaken special training in bilingualism or autism. All participant SLPs at least knew 3 or more languages and were thus multilingual.

**Table 1:** Demographic details of the participant SLPs.

Participant ID	Years of experience	Place of practice	Work setup	No. of languages known	First and Second Language(s)
P1	6-10	Goa	Private	3	Konkani, English
P2	11-15	Mangalore	Institutional	4	Malayalam, English
P3	2-5	Gurgaon	Institutional	3	Marathi, English
P4	11-15	Bangalore	NGO	5	Kannada, English
P5	11-15	Lucknow	Private	3	Hindi, English
P6	2-5	Mysore	Institutional	3	Kannada, English
P7	11-15	Mysore	Institutional	3	Telugu, English
P8	2-5	Trivandrum	Private	4	Malayalam, English
P9	6-10	Mangalore	Private	6	Kannada, English
P10	6-10	Delhi	Institutional	3	Hindi, English
P11	11-15	Mangalore	Private	5	Punjabi, English
P12	6-10	Bangalore	Private	4	Kannada, English
P13	11-15	Bangalore	Private	4	Hindi, English

Analyses of the data revealed five major themes describing SLPs’ attitudes on practices in children with autism having bilingual exposure: (1) SLP’s opinion of monolingual vs. bilingual approaches in children with autism, (2) Bilingual practices for children with autism, (3) Factors explaining the choice of language(s), (4) Suggestions for parents of children with autism and future SLPs, and (5) Need for bilingual SLP practice. Table 2 displays the list of main themes and corresponding sub-themes. Results for each theme and sub-theme is presented below with respective frequencies of response from the participants.

**Table 2:** Overview of themes and subthemes.

Theme	Subthemes
SLP’s opinion of monolingual vs. bilingual approaches in children with autism	India, a multilingual country
	Advantages vs. Disadvantages of bilingualism
	SLP’s opinion on monolingual approach for children with autism
	SLP’s opinion on the bilingual approach for children with autism
	Bilingualism helps in language transfer
	Difficulty learning a second language due to limited exposure
Bilingual Practices for children with autism	Communication assessment in bilingual children
	SLPs’ practices in a less proficient language
	Sequential vs. simultaneous bilingual approach in therapy
	Focus on communication skills over linguistic diversity
	Parental concerns and experiences with a bilingual approach
Factors explaining the choice of language	Preference for English
	Relevance of the child’s native language
	During early intervention
	Social and cultural background
	Autism severity and associated conditions
	Choice of language for school-going children
	The language of local community
	Communication partner
Suggestions for parents of children with autism and future SLPs	Advice parents to avoid misconceptions about bilingual exposure
	Advice for future SLPs
Need and scope for bilingual practice	Need for standardized tools for assessment
	Importance of proficiency in more languages for SLP
	Need for further research and practice guidelines

### SLP’s opinion of monolingual vs. bilingual approaches in children with autism

This theme reflected the SLP’s attitude regarding monolingual and bilingual approaches during their practice on children with autism. Six sub-themes emerged from this theme, explained as follows:

#### India, a multilingual country.

SLPs (3 of 13 participants, 23%) stated that it is rare to find children in India who have been exposed to only one language, and it is typical for everyone to have encountered multiple languages. In India, bilingualism is inevitable.

A few of the SLPs (2 of 13, 15%), from different regions of the country and different work settings, stated that every child must be exposed to more than one language to adapt to the Indian educational system. This sub-theme reflects the need for bilingualism in Indian cultural as well as educational context.

*“Bilingualism,.. it is a fact of life… especially in India every child is exposed to minimum two languages… therefore therapy should be done in both the languages” (P5)*

#### Advantages vs. disadvantages of bilingualism.

Some of the SLPs (5 of 13, 38%) across distinct regions and work settings, demonstrated a positive attitude towards bilingualism. They stated that bilingualism benefits and encourages children’s cognitive and language development (P2, P5, P6, P8, P13). A few other participants (3 of 13, 23%), with more years of experience further stated that bilingualism promotes communication flexibility (P2, P5, P12).

*“In general, bilingualism, for that matter... more than one language helps in language learning. We know children are capable of learning multiple languages. I have read somewhere, and especially during their critical period if they’re exposed to many languages, you can develop their cognition and functions like the language functions, and it does help them communicate better then”. (P5)*

While concerns were raised by one of the participants (P2) having more than 10 years of experience, about a child’s ability to achieve proficiency in both languages. This sub-theme reflects that while SLPs are knowledgeable and agree on the benefits of bilingualism, a few SLPs do believe that bilingualism could be demanding for children with ASD.

*“Bilingualism can impact the child negatively because he may not be proficient as he has to use two languages, but at the same time we may find that he may not be so comfortable speaking in the language which he may not be proficient.” (P2)*

#### SLP’s opinion on monolingual approach for children with autism.

A couple of participants (2 of 13, 15%) from distinct regions and work settings stated that the monolingual approach could be beneficial during the child’s early years but may not be for school-going children (P7, P3).

*“I think exposing them to one language is better than exposing them to two languages in the early years.” (P3)*

Further, a few of the experienced SLPs (4 of 13, 30%) across regions and work settings stated that children with ASD towards the severe side of the spectrum, benefit more through the monolingual approach (P1, P2, P7, P13).

*“If the child is on the more severe end of the spectrum, then the parents might have to stick to one language while speaking to the child, so that it doesn’t stress the child out.” (P1)*

Some of the SLPs (3 of 13, 23%) committed that the monolingual approach might be good for children with autism but may not be appropriate in the Indian scenario. One of the experienced SLP stated that monolingual approach could be followed based on the parent’s choice or the child’s response (P7). Thus, this sub-theme reflects that SLPs believe that monolingual approach works better for children with ASD in the severe end of the spectrum in the initial phase of communication intervention. Although, a monolingual approach may not be appropriate, given the predominant bilingual home and community contexts in India and thus should be considered on case-to-case basis upon discussion with the parent/caregivers.

#### SLP’s opinion on the bilingual approach for children with autism.

Some of the SLPs (3 of 13, 23%), from the southern India, stated that the outcome from dual-language exposure for the child is individualistic.

“*Each child learns very differently, so if a child is capable of learning two languages, then I will encourage, it is individualistic… if the child is learning two languages, it is good for the child because it improves communication, whatever advantages are reported for bilingualism can be same for autistic children.” (P7)*

A few other SLPs (2 of 13, 15%) reported that the success of bilingual approach would depend on various factors, such as the child’s age, socioeconomic status, communication needs, etc. Other SLPs (4 of 13, 30%) across different regions and work settings stated that the bilingual approach helps to improve pragmatic and language skills, which promotes communication and social interaction (P5, P7, P6, P12).

*“the languages which we speak, we also use a lot of gestural language with the children… so using a gesture especially for a child verbally in the spectrum would only boost their knowledge in each language or both, boost their vocabulary… I don’t think putting them down in any of the area, in any type of development, especially with speech and language development. I think introducing 2 languages should always be an advantage” (P8)*

One of the experienced SLPs stated that the bilingual approach helps high functioning children with autism and helps them to improve their school and academic performances (P7). Another SLP from private work setting stated that exposing a child to two languages is not only beneficial but helps boost child’s knowledge and stimulates both languages (P8). Some of the SLPs (2 of 13, 15%) stated that being bilingual is required in today’s community.

This sub-theme highlighted cultural and geographical differences in SLPs’ beliefs and practice on children with ASD. As bilingualism is more prevalent in southern India, SLPs from southern regions provided insights on their bilingual practice with suggestions on candidacy for children on the ASD spectrum. Most SLPs irrespective of their respective regions, believed that bilingual practice would better support the communication and social interaction in autism.

#### Bilingualism helps in language transfer.

SLPs (4 of 13, 30%) reported that switching between languages is common in typically developing children when they are exposed to two or more languages, which in turn, enhances communication flexibility and supports social and communication development. They believed that it is also true for children with autism. These SLPs were from southern regions and worked exclusively with autistic children, suggesting positive attitude for bilingual practice.

*“I think so because we always learn from typical development in children. There is a theory called the transfer of language. For example, if you are learning Malayalam as first language and then learning English as a second language, then there is always the transfer of, you know, the vocabulary. I believe this happens in children with autism also… so when you have an L1 and L2, something else you know… that, I feel that it will only compliment it”. (P8)*

#### Difficulty learning a second language due to limited exposure.

A few of the SLPs (2 of 13, 15%) from distinct regions and different work settings believed that delays in learning of first language makes it difficult for children to learn a second language as well, owing to more exposure to the first language. This sub-theme highlights the knowledge of SLPs that language delay could impact learning of second language by limiting the input.

*“even though the child was exposed to most of the times in the home, the native language or the first language the child has delay, second language is something which has not been exposed for the most of the time how first language has been exposed so it is definitely difficult task to achieve the second language”. (P6)*

### Bilingual practices for autism children

This theme reflects the methods used by the SLPs to conduct the assessment and intervention processes in bilingual children with autism in the Indian context. The following sub-themes emerged from this theme.

#### Communication assessment in bilingual children.

Most of the SLPs (5 of 13, 38%) across regions and work settings stated that assessment should be carried in all the languages that the child is exposed to and that they try to perform assessment in all the languages child is exposed to (P5, P7, P11, P12, P8).

*“If one language is affected, probably there would be a problem with the other language too … but it’s not a hard and fast rule, what I see in one child would happen again or another child too. We should perform the detailed assessment in both languages and then check”. (P11)*

One of the experienced SLPs from northern region stated that she carries out the assessment informally based on the observation and then plan the intervention accordingly (P5). A few other SLPs (3 of 13, 23%) all from the southern cities stated that they would use available standardized test materials for assessment of autism, and in addition to that, they would use language assessment scales to check for language development (P2, P4, P13). P2, an experienced SLP stated that the resources need to be child-specific, and the materials to be chosen to depend on the child’s need and the severity of autism.

*“it doesn’t have to depend upon their language background of the child …so their resources are purely based upon the need of the child, communication level, and of course based on the severity of autism, you will decide what type of resources… so I can’t list out what are the types…yes, it totally depends upon the need of the children type of severity or type language level of the child.” (P2)*

One of the experienced SLPs added that psychological evaluation and communication evaluation is crucial to know the child’s abilities, which helps to plan a detailed and structured intervention plan (P4). This sub-theme reflected that SLPs choice of assessment practices differed by geographical location. Since bilingualism is more frequent in southern parts of the country, there is better availability of standardized tools in languages spoken in south, for instance, Kannada, would have influenced SLPs use of standardized tools for assessment. SLPs also highlighted the need for adaptive assessment practices to address the needs of children with autism.

#### SLP practices in a less proficient language.

Most of the SLPs (5 of 13, 38%) across regions and work settings stated that they were not very confident in using a language for assessment or intervention that they learned later in life, especially as a part of their academic course. Almost all SLPs (11 of 13, 84%) across regions and work settings reported that when they are not proficient in the child’s native language, they would require help from a native speaker of the child’s language, preferably a family member, to be a translator during the early intervention.

*“In the initial therapy, I will make mother sit inside the session and take therapy in her presence, okay… because the language spoken at home should be the language that should be used in the initial days of therapy.” (P9)*

Another SLP, who worked in region where English was prevalent stated that she would refer the child to fellow clinicians who speak the child’s native language to avoid relying on the parents or others during the intervention (P1).

In contrast, some participants (2 of 13, 15%) stated that they could carry out interventions without help, even with less proficiency, although they find it tough. Few other experienced SLPs (3 of 13, 23%) across regions believed that proficiency in a language is not mandatory to provide services for children with autism (P7, P9, P5).

Other SLPs (3 of 13, 23%) across work setting from southern cities stated that they try and learn the child’s native language, its content and function words initially, and then switch to English over time, especially for intervention (P6, P13, P8).

One of the SLPs from a northern (urban) city stated that the effect of language proficiency depends on the geographical location, and most parents at her location prefer English; hence she doesn’t have to use other languages during her practice (P3). Another experienced SLP stated that she could manage her practice in client’s language despite her limited proficiency as the children with ASD in her clinics were mostly nonverbal or minimally verbal.

*“I am confident in my practice, as most children with autism are nonverbal. Only the basic proficiency in the language is required to carry out therapy”. (P9)*

This sub-theme therefore revealed that SLPs practice in less proficient or non-dominant languages varies by geographical location, experience and client abilities.

#### Sequential vs. simultaneous bilingual approach in therapy.

A few SLPs (5 of 13, 33%) across regions and work setting believed that for children with autism, it is advisable to focus on one language until they are proficient in that language. They further felt that it is better to focus on the language in which the child has good comprehension skills. A few of the SLPs (2 of 13, 15%) stated that since children with autism have delayed language skills, they should be exposed to one language to avoid confusion, thus suggesting a monolingual approach.

***“*** *Initially, it is better to be consistent with one language only because, first of all, the language confusion matters, but again I would say it depends on the severity of the autism.” (P9)*

One of the SLPs from a northern city stated that the child should be exposed to one language, generalize it and strengthen the vocabulary in that language before exposing the child to a second language. Few of the SLPs (3 of 13, 23%) from southern regions and varied experience stated that once the prelinguistic skills are achieved, a second language can be introduced if the child has good receptive skills in one language (P6, P10, P13). An SLP believed that the intervention in one language facilitates development in the second language.

One SLP stated that autism severity and language abilities are not always correlated, but it is better to start with one language, and once the child has good comprehension, then can introduce the second language. Two SLPs stated that using two languages simultaneously may confuse children with autism with language delays.

*“The first thing is simultaneous exposure. We usually do not recommend it because it might create confusion because already the child is not exposed to what has to be exposed, but if you start giving two inputs or two languages like they will have the confusion... what to take and what not to..okay… which might lead to another problem so that will be again a challenging task for SLP as what to take off or anything… we don’t know this …so there must be a mislearning as well or confusion will be there”. (P6)*

SLPs (4 of 13, 30%) with varied experience and work settings stated that they would suggest parents do simultaneous exposure to two languages and stimulate in both languages as it promotes communication flexibility. While a few of the SLPs (2 of 13, 15%) stated that whenever a child is exposed to two languages, based on their pros and cons, one language should be selected without affecting the other.

As stated by another few experienced SLPs, (2 of 13, 15%) if a child is exposed to two languages and has better expressive skills in one than the other, both languages should be stimulated to improve communication interaction.

*“If a child is being exposed to and understands two languages, but talks more in one language in that case, both languages should be focused and encourage the child to use both languages… then only it improves child’s ability to communicate in both.” (P10)*

Taken together this sub-theme implies that most SLPs favoured sequential bilingual exposure for children with ASD. Nevertheless, SLPs differed in their practice of use of sequential or simultaneous bilingual approach based on their experience and child characteristics such as severity, prelinguistic and comprehension skills.

#### Focus on communication skills over linguistic diversity.

A few of the experienced SLPs (3 of 13, 23%) from different regions expressed that communication should be prioritized over language ability and the number of languages children speak on the spectrum. One of the SLPs stated that functional communication should be achieved in all the languages the child speaks (P13). Another SLP stated that functional communication in the native language helps the child in social situations (P1).

*“maybe in some situations, the child will miss out on social situations which he/she won’t understand … at least functional communication in those places, you know… because everyone… for example, if you go to a family function everyone will be speaking in their native language, and the child is lost … you do not want the child to feel that way… and first of all they have social communication issue and then on top of that they aren’t able to communicate in that social environment.” (P1)*

A few other experienced SLPs (2 of 13, 15%) stated that pre-linguistic skills and communication skills should be focused more on children with autism as they are poor in communication and social interaction skills. This sub-theme thus reflects SLPs gave emphasis on functional communication in native and other language(s) child is exposed to. This was based on the challenges in social interaction experienced by children with ASD.

#### Parental concerns and experiences with a bilingual approach.

SLPs (2 of 13, 15%) practising in different regions reported that most parents believe exposure to two languages is a mistake.

“*What I have noticed is once a child comes to me, generally they are around maybe 2 or 3 years of age, and once they are diagnosed…also you know that by that time the child is already introduced to both languages… mostly the parents ask, if they made a mistake by introducing both the languages… which, I generally tell them is that, not all you know.. they have not made a mistake, it is fine, and the child is capable of picking up both… but they can decide”. (P1)*

An SLP experienced parents switching from their native language to English upon advice received from other professionals, which could be stressful for the child. A few of the SLPs (2 of 13, 15%) stated that parents do raise concerns that switching to a less proficient language becomes tough and stressful, as the family may not speak English.

Few SLPs expressed (2 of 13, 15%) concerns over the fact that parents use a less proficient language and stimulate the child with the same, it might lead to the child’s mislearning of the language.

*“when parents share their concern, their main concern is that they don’t know much of English how will they teach the child… that is what is the main concern.” (P4)*

Some other SLPs (2 of 13, 15%) stated that they would not advice the parents to speak in a less proficient language, rather use one language which is spoken at home. This sub-theme suggests the importance of parental and home language background considered in SLPs practice.

### Factors explaining the choice of language

This theme presents the factors guiding the SLPs in choosing the language for interventions. Eight sub-themes emerged from this theme, presented as follows:

#### Preference for English.

Most of the SLPs (7 of 13, 53%) across regions and work settings stated that nearly all parents preferred the bilingual approach, which includes English, as it helps with schooling and English becomes the predominant language in the later stages of a child’s career.

“*What I have noticed is when the child goes to school, English becomes the more predominant language, and many of the children only speak their native language.. may be in one word or two words, you know… I have noticed that their expressive language is poor in their native language when they join their school.. okay… English will become more dominant right that’s what I have noticed.” (P1)*

A few of the experienced SLPs (3 of 13, 23%) reported that English also becomes important as they use it for reading and writing, whereas the native language is mostly used for only communication. This sub-theme thus reflects the need for English inclusion and thus bilingual practice in Indian context.

#### Relevance of the child’s native language.

Many SLPs (6 of 13, 46%) across regions and experience stated that if the child is exposed only to the native language, knowing it becomes very crucial. A few experienced SLPs (3 of 13, 23%) stated that child’s proficiency in the native language facilitates the intervention process and helps to focus more on improving vocabulary and prelinguistic skills.

*“definitely if you are more fluent in their native language, of course the progress will be better and the child will learn better.” (P1)*

Other SLPs (4 of 13, 30%) with varied experience and work settings believed that knowing a child’s native language is crucial for rapport building with the child (P8, P1, P13, P5).

An SLP further stated that it is important to know all the languages the child is exposed to in all communicative environments, including digital media (P5).

*“It’s very important for us to know what kind of environment child is in, what kind of spoken language and also the digital language that the child is exposed to… because you know many a times we and children are exposed to… the language exposure is more from the digital media… so understanding the language exposure of verbal language in the environment including the digital media.” (P5)*

This sub-theme reflected SLPs emphasis placed on the native language of children with ASD, irrespective of their work setting and regions.

#### During early intervention.

SLPs expressed (2 of 13, 15%) their opinion that, during early years, if the child is confused, it’s better to stick to one language, especially if the child has other associated conditions.

“*definitely, see exposing the child to multiple languages at early years is a slight risk actually, if the child is showing the lag and switch language ability and when there is an associated condition such as autism… then, in that case, I think sticking to one language and functional vocabulary…working on the functional vocabulary of one particular language is more beneficial than.. you know… teaching two languages at a time …I might be wrong… I think exposing them to one language is better than exposing them to two languages during the early years.” (P3)*

One of the SLPs reported that using one language is preferable during early intervention if parents speak two languages (P2). A few other SLPs (4 of 13, 30%) across regions and work settings stated that through early intervention, we could engage the parent in therapy, teach them techniques, and subsequently generalize to all communication environments (P4, P1, P3, P7). Furthermore, some SLPs (2 of 13, 15%) felt that it’s better to start early intervention in the mother tongue of the child and slowly introduce the second language. In contrast, another SLP from southern region believed that there is no age to start exposing the child to two languages. As language is a precursor for literacy skills, exposure to two languages is advisable as early as possible (P8).

This sub-theme reflects that most SLPs recommend and practice home language for children with ASD, in the initial phase of intervention. While a few others believe that two or more languages should be introduced to children with ASD from the beginning.

#### Social and cultural background.

Some of the SLPs (3 of 13, 23%) working in southern (urban) cities stated that English is the choice of language for families with higher socioeconomic status, and the choice of intervention should be individual specific depending on the cultural background.

*“so …most of the parents whom I have seen are from well-to-do families speaking English very well… so they need not switch the language because most of them were already using English.” (P12)*

Another SLP too stated that bilingualism could benefit children based on their age, socioeconomic status, and communication needs (P6). Thus, this sub-theme reflected the importance of social cultural backgrounds of the clients in deciding the bilingual approach for children with ASD.

#### Autism severity and associated conditions.

Some SLPs (3 of 13, 23%) mentioned that the child’s prelinguistic, receptive language and language learning capabilities matters in selection of language, not severity. While other SLPs (2 of 13, 15%) stated that it would be hard for the child with severe autism and associated conditions, and that they may struggle to learn two languages.

“*The more severe the autism, the more difficult they will find to comprehend… when exposed to two or more languages.” (P2)*

Another SLP, in contrast, stated that it’s not always difficult for the child to learn a second language, even with severe autism (P10).

Many SLPs (4 of 13, 30%) across regions and work settings believed that autism is a heterogeneous condition. If the child can pick two languages, we encourage it, if not, we stick to one, and the child’s improvement depends not on language but many other factors.

*“so I would not say that they will not be able to pick up the language… but what happens is that, the sensory issues that might make it diffic*ult for them to learn the expre*ssive* language… it is j*ust easier for the parents to stick to one language… and then the child picks it up… that is what I have noticed.” (P1)*

This sub-theme revealed the heterogenous profile of children with ASD and hence, the choice of language varied across SLPs based on autism severity and other factors.

#### Choice of language for school-going children.

SLPs (5 of 13, 38%) from southern regions and institutional work settings stated that it is better to stick to the child’s school language or the medium of instruction, as they use it for reading and writing purposes. On similar lines, few other SLPs (2 of 13, 15%) opined that it’s typical for school children to get exposed to at least two languages. Hence, they would choose a bilingual approach.

*“I would recommend them to go with a bilingual approach and ask them to stimulate in both languages. Most often, I see that the child is exposed to a second language at school. That is a very typical situation… but when we get children below four and all, then it varies”. (P7)*

In contrast, another SLP working in private clinics reported that the child’s reading and writing ability should determine the school’s medium of instruction, and if the child can read and write in his/her native language, they would recommend a same-medium school (P4). This sub-theme showed difference in SLPs approach in choice of language for children with ASD by work setting.

#### The language of local community.

A few of the SLPs (3 of 13, 23%) working in private clinics mentioned that the language choice should consider the local/community language of the region from where the child comes from. The SLPs argued that the majority language environment would help the child to communicate in the long run, more than the number of languages the child is exposed to.

*“I think it is a good practice if the language chosen is the language of the community, where the child is supposed to... you know… stay in the long run … like the years to come.. for example he is in Mangalore and the parents know that the child will always stay in Mangalore then even if he learns Kannada it is cool but if the child is in Karnataka for a small period of time and then moving to maybe north Indian city, then I think in that case using a language which is either bilingual or the child should be exposed to a regional language or the language which is common to both the places or either hindi or english probably.” (P3)*

This sub-theme reflected the view of SLPs to consider community/societal language for children with ASD.

#### Communication partner.

Participant SLPs (2 of 13, 15%) with experience stated that they initiate language stimulation in one language considering the communication partners’ preferred language, and depending on the child’s potential, introduce a second language. A few other SLPs (3 of 13, 23%) also recommended that the choice of language for a child with autism should depend on the communication partner of the child. For instance, grandparents might not know English, which would affect the child’s interaction with them owing to language barrier.

*“there are parents who come up and say SLPs and special educators and others advised to use only one language at home, so that the child doesn’t become confused but that’s a burden for the parent… because probably the therapist are totally well versed in English, so the parents will use… but what about the grandparents… they also have to share a lot of things with children …so that’s …I think that’s a burden for the parents… I have come across such situations.” (P10)*

The sub-theme reveals the importance of considering language used with communication partner(s) on a day-to-day basis.

### Suggestions for parents of children with autism and future SLPs

This theme presents SLPs advice and suggestions to the parents of children with autism and future SLPs regarding bilingual practice. Following subthemes were observed. While most but not all SLPs discussed this theme.

#### Advice parents to avoid misconceptions about bilingual exposure.

SLPs across regions and work settings reportedly (6 of 13, 46%) advised parents to avoid misconceptions about bilingual exposure using counselling, parent interaction, and providing evidence based literature. One of the SLPs suggested encouraging parents to attend workshops to improve their knowledge and get updated with recent advances regarding bilingualism in autism.

*“I make parents talk to other parents who have been through the therapy..okay, for example… I have sessions … I have parents coming up who have older children diagnosed with autism and who have been through the phase of using 2 languages at the same time… so I personally make the parents talk to the other parents so that, you know… they get confidence that, okay using two languages would not be a harm… and also I do tell them about the research that is going on right now… what is the research change about …so I give them research evidence also and also you make them talk to the parents, because more than therapists they find confidence in parents who have gone through it already.” (P8)*

A few of the other SLPs (3 of 13, 23%) from northern regions recommended parents to switch to child’s preferred language and enhance their proficiency in that language to avoid grammatical errors, as stimulation of the language at home is crucial.

*“see sometimes parents have already switched to second language so I can only suggest them to work on their grammar and ask them to kind of learn the language proficiently in a better way learn together grammatical mistakes and all are not observed in the child’s language okay yes but if the parents ask me shall we switch to second language for the sake of the child because the child is exposed to more of a digital media in English and the child speaks only in English a few phases are in English just like contextually relevant then I would say switch to English because the child is comfortable with.” (P5)*

This sub-theme revealed that SLPs used evidence-based bilingual intervention practice for children with ASD and educate parents against misconceptions related to bilingualism.

#### Advice for future SLPs.

Most SLPs (7 of 13, 53%) across regions and work settings stated that evidence-based practice is crucial, and that SLPs should be well updated with recent research literature, attend workshops, to update their knowledge in the field. Other SLPs (4 of 13, 30%) suggested that planning goals and activities for intervention is important, including cognitive and behavioural aspects along with goals for language.

A few of the experienced SLPs (3 of 13, 23%) affirmed that autism is a heterogeneous condition, and so the intervention should be individual-specific, that should not underestimate a child’s abilities.

*“ It is not right that you are not exposing the child to another language… you cannot presume and decide that a child with autism will never learn the second language… you are underestimating a child.” (P10)*

“*It will be a very individual thing. Probably one child could pick it up and get better at one language, or can communicate better, but for another language, another child, it would turn out to be a challenge. It should be a parameter which is, you know, which depends on individual to individual… not very sure how this could turn out”. (P11)*

One of the SLPs recommended that in Indian context, it’s necessary to do the communication evaluation and intervention in all the languages the child is exposed to. This sub-theme highlighted SLPs advice for adoption of a comprehensive tailor-made approach for ASD based on child’s profile and learning capacity.

### Need and scope for bilingual practice

This theme presents the need and scope for bilingual practice and consisted of following three subthemes. Most but not all SLPs discussed this theme.

#### Need for standardized tools for assessment.

Many SLPs (6 of 13, 46%) across regions and work settings highlighted issues such as the need for standardized tools and test materials to measure language skills in bilingual and multilingual population. The SLPs reported (4 of 13, 30%) that either developed test materials are not available or accessible, or there is a need to update the normative for such tools in more languages.

“*well… I’ll be honest there are not much you know specific tests which are available for bilingual clients or multi language maybe general ones which are being used if your talking about autism if you know ComDEALL checklist or you know general ones like 3DLAT or all those all those once which are used or not specifically meant for bilinguals its general ones so I don’t see in fact I would say there is no specific test at all the which is specifically for bilingual.” (P13)*

This sub-theme reflects the lack of standardized tests and norms for bilingual children in Indian context.

#### Importance of proficiency in more languages for SLP.

Many SLPs (6 of 13, 46%) with varying years of experience argued that SLPs in India would benefit from knowing more languages by providing better services to culturally and linguistically diverse clientele.

*“You can actually help a lot more clients, you know… in those languages that you are fluent... like maybe… you can do parent training and things like that…if you are fluent only in a particular language, then the help you give will be only limited… and actually taking intervention for a child, in that case would be tough.” (P1)*

Nearly half of the SLPs (7 of 13, 53%) across regions and work settings mentioned that it is crucial for SLPs to know the regional language as well as the dialect at their workplace. A few of the SLPs (4 of 13, 30%) felt that SLPs should know languages at least for purpose of encouraging functional communication. This sub-theme reveals the necessity for SLPs to be bi/multilingual in Indian context.

#### Need for further research and practice guidelines.

Almost half of the SLPs (7 of 13, 53%) expressed the need for guidelines to practice with bilingual children. They further voiced the need for the assessment protocols, and more research in the domain of autism and bilingualism.

*“I think assessment protocols really need to get revised …you know… we really need a lot of things which focus on these kind of areas like the bilingual population… and then probably beyond that, for intervention strategies …we can get a scenario where a lot of children are bilinguals so what exactly should be the right approach to be dealt with …see, what we are doing is we are all doing things out of our experience and knowledge… so, it’s all individual specific… probably this is my viewpoint and then any other SLP is going to have another viewpoint… so if we can have something you know which gives us the right kind of guidelines on bilingual children so it would help as how you are supposed to go about it.” (P11)*

This sub-theme identified the lack of need for practice guidelines for bilingual children with ASD.

## Discussion

Against the limited research evidence and a lack of knowledge among professionals regarding practices in bilingual children with developmental disabilities, present study aimed to investigate SLPs’ attitudes and practices towards assessment and intervention of children with autism having bilingual exposure for the first time in the Indian context. The study employed a qualitative in-depth interview method. Results of the study revealed mixed attitudes and practices in SLPs regarding the bilingual approach towards assessment and intervention of children with autism in India. The identified themes and corresponding sub-themes offer a broader understanding of the SLPs’ perspectives on bilingualism and their practice and choice of language for assessment and intervention in children with autism, which is one of the long-standing needs in the Indian multilingual context. All SLP participants in the present study were multilingual and worked in various settings and their caseload consisted of children with autism. Below we present an overview of the SLPs’ language background followed by a discussion of findings from the present study.

All SLP participants in our study were multilingual, unlike in other international studies, where most SLPs were monolingual [23,35]. An international survey conducted regarding language intervention for multilingual language-impaired children on 99 SLPs from thirteen countries showed that most (74%) SLPs were monolingual [36]. Language status, thus, did not pose difficulty for SLPs working with the predominant multilingual caseload in the present study. Most of the languages spoken by the SLPs in the present study matched those spoken by children in schools and communities (viz., English, Kannada, Hindi, Malayalam), similar to the findings of another study on SLPs working in Australian schools [37]. For a few of the local languages spoken by some children in SLPs’ caseload (e.g., Tulu, Konkani, Baery) in our study, only a few SLPs possessed minimal to functional proficiency levels. As a multilingual country, it is rare to find children in India who are only exposed to just one language. It is rather typical for everyone to have encountered multiple languages and dialects, especially in urban areas. In India, bilingualism is inevitable [38]. Besides, every child is exposed to more than one language at school due to the Indian educational system that follows the three languages system [39]. This finding thus necessitates multilingual competence and practice for SLPs working for children with ASD in the Indian context.

Many SLPs in our study (7 of 13, 53%) showed mixed attitudes towards bilingualism in children with autism, similar to previous literature [40,41]. It is important to note that SLPs cultural attitude towards bi/multilingualism can impact parental and SLP decision-making [42]. For instance, parents with higher SES/more financial stability in UK choose bi/multilingual environment for their autistic child. Findings of our study reveal attitudes of SLPs from a socio-linguistically diverse setting as India, which is distinct from other contexts (monolingual and economically advanced) in available studies. Similar to the UK study, we found that SLPs attitude towards bilingualism differed by geographical and cultural differences within India. Those SLPs supporting the bilingual approach for children with autism reported that language for assessment and intervention should be decided on a case-to-case basis according to the child’s linguistic abilities and autism severity. While others recommended a monolingual approach for autism, during the early years, and a sequential approach until they learn one language, for children in the severe end of the spectrum. This finding is on par with the literature indicating that the type of approach for intervention should depend on the linguistic characteristics of the child [43]. Results of our study, thus, recommend development of framework for assessment and intervention of children with language disorders exposed to bi/multilingual environments, which could help SLPs in decision making. These guidelines should carefully consider the heterogeneity and associated issues reported in children with ASD. There also is a need for separate guidelines for practicing SLPs and parents of bi/multilingual children with ASD. SLPs in our study opined that bilingualism helps in language transfer and thereby facilitate communication. Although language transfer phenomenon is commonly reported in typically developing bilingual children [44], its impact on language development in children with autism is not known [45].

SLPs’ practices on bilingual families with autism in our study again showed mixed results, i.e., the use of both bilingual as well as monolingual approaches for assessment and intervention services. SLPs practice in the present study differed by geographical location, SLPs’ experience and client needs. Bi/multilingual was more common in children and families from southern regions and urban cities, given the socio-economic differences and educational policies in schools with English medium of instruction. SLPs working in southern regions more often used bi/multilingual assessments as standardized tools were available. Our findings thus have implications for development of language tools and protocols to facilitate bi/multilingual assessments in India. SLPs made use of available resources, viz., tools and translators as parents or colleagues during their practice. A few SLPs (4 of 13, 30%) believed focusing on communication training over language and discussed parental experiences influencing their practice. Nearly one half of the SLPs (7 of 13, 53%) explained using a sequential bilingual approach (of exposing the two languages) for language intervention in children with autism, given limitations in the child’s cognitive and linguistic abilities. While other SLPs (4 of 13, 30%) believed that simultaneous bilingual exposure of the two languages better support communication flexibility for children with autism and should be thus encouraged. These findings align with the earlier reports of differences and variabilities in opinion and practice of SLPs working with children having communication disorders [46]. SLPs discussed about parental concerns and experiences with bilingual approach, where the parents felt making a mistake by exposing their child with autism to two languages. SLPs reported parents switching to monolingual approach and using English (language in which they are often not proficient) instead of native language, which was very stressful. SLPs reported using parental education and counselling in their practice to address these issues. Our findings, thus, call for the need of creating awareness regarding bilingualism in parents and healthcare professionals [47,48]. Parents could be educated about bilingualism by creating awareness through public education platforms such as social media, newspapers, podcasts and workshops. SLPs could be prepared by training institutions and governing councils modifying the existing curriculum by including evidence-based approaches for assessment and management of bi/multilingual children with autism. SLPs in the study also emphasized on use of parental and home language in their practice which aligns with the current practice guidelines [27,30].

Our results on factors explaining language choice showed participants consideration for promoting English as well as the native language of the child. While SLPs acknowledged the importance of intervention in the child’s native language, they also reflected the need for teaching English, as the common language for the child’s schooling and training. This finding aligns with studies where parents of children with autism expressed the need for formal education to be conducted in English rather than in their native language [47–49]. They believed formal English instruction would support their academic performance, open more career options, and make their livelihood easier [50]. This finding, therefore, points to the predominance of English in the Indian educational set up as an important factor determining language choice [47]. Besides, English is also preferred as a common language in the Indian context given the number of languages and dialects of languages spoken by the community [51]. SLPs also stated the relevance of training in the native language along with English, considering factors such as early intervention, socio-cultural background and geographical area the families come from, autism severity and the associated conditions, and communication partner preference. These factors are similar to the reported factors influencing language selection in children with autism in Indian context [48]. SLPs stated that families’ socio-cultural background influenced their decision of selecting the language of intervention given parental ease of learning/using English and the community language being same or different than their native language. SLPs’ opinion on choice of language of intervention for school-going children with autism was rather divided. A few of the SLPs suggested use of English given the common language for schooling as well as intervention, other SLPs proposed use of bilingual approach as school system follows a bi/multilingual instruction. SLPs considered age of the child in proposing these choices by recommending choice of language of instruction for school aged children, while native language for younger children with autism. SLPs in our study also cautioned about the risk involved here as some parents might not be very proficient in English, while they tend to use the same language with the child. Using a language with less proficiency with children might lead to children mislearning a language, and reports show that a communication partner’s poor proficiency in a language during a conversation with the child, can have a detrimental effect on both the child’s learning and the family–child communication [16]. Some of the SLPs also emphasized on including community or societal language for communication and intervention to facilitate social interaction in children with ASD. These findings thus, point towards the need for development of guidelines and policies for bilingual families and children with autism specific to Indian context [48]. Such guidelines could specifically address assessment in understudied languages in India using non-standardized methods such as language profiling, dynamic assessment, criterion reference and processing dependent measures [30]. Our findings also recommend suggestions for policymakers to consider providing infrastructure and facilities to schools to recruit and provide bi/multilingual SLPs in special schools and hospitals and centres catering to children with ASD in India.

Findings of our study offer suggestions for the parents of children with autism in terms of avoiding misconceptions on bilingual exposure and supporting their child’s language learning. Studies on parental views on the bilingual approach indicate that parents of children with autism express greater concern regarding bilingualism than parents of typically developing children, often worrying that multiple languages may confuse their child and lead to a further delay in language development [16,47–49]. SLPs in the present study suggest overcoming this issue through parent education, discussions with fellow parents, and parental counselling. Our findings offer suggestions for SLPs by updating the knowledge of evidence-based practice, and by adopting individualized intervention for children with autism. Our results also highlight the need and scope for bilingual practice in the Indian context, such as the development of framework or tools for assessment in multiple Indian languages, the need for SLPs to be multilingual and the need for future research and development of practice guidelines for children with autism [32,48,52].

Findings of our study provide a descriptive account of SLPs’ views regarding beliefs on bilingual practices in children with autism from multilingual Indian context. Besides, participants in our study were experienced SLPs working in varied setups and different regions of the country. Results of the study further revealed factors influencing choice of language and bilingual practices relevant to the Indian context. Our findings, therefore, provide SLPs experiences and views from a multicultural and multilingual Indian context, distinct from the predominant monolingual western cultural context. The study findings, therefore, add to the literature of evidence-based practice in bilingual children with ASD and offer specific implications for SLPs, speech pathology training institutions, parents and policy makers for working with bi/multilingual children with ASD. The study had a few limitations as well. Firstly, most of the participants (8 of 13, 61%) in the present study belonged to the southern parts of India, although SLPs from different regions of India were approached. The findings of the study, thus, cannot be generalized to SLPs practicing in India as a whole. SLPs in this region generally have a higher degree of exposure to multilingualism compared to other regions. The availability of standardized language tests is also not uniform across languages and regions in India. These factors could have affected their attitudes and practices towards bilingual approach. Another limitation was relatively small sample size of SLPs recruited in the study. While a data saturation was observed by 10^th^ participant, more participants representing different regions and cultural backgrounds could have provided further insights to the study. There is also a possibility of self-selection bias in the SLPs recruited, that could have influenced the findings of the study. Future studies could therefore be conducted on larger and diverse sample of SLPs with representations across the regions of India to cover the wide socio-linguistic diversity.

## Conclusion

There is increasing need and growing interest for research exploring knowledge, beliefs, and practices of SLPs regarding bilingualism in children with autism. Hence, the present study aimed to investigate SLPs’ attitude and practices related to the assessment and intervention of children with autism having bilingual exposure in the Indian context. An in-depth interview was used to collect data from 13 experienced SLPs working across India in different setups. All SLPs were multilingual in our study and had experience in working with autistic children. Study findings revealed mixed attitudes and practices regarding the bilingual approach towards assessment and intervention of children with autism, that varied with geographical and cultural differences among SLPs practicing in India. SLPs discussed various factors for consideration of language choice, based on child’s profile along with the suggestions for parents of children with autism and fellow SLPs. They also emphasized the need and scope for bilingual practice in India. These findings provide a detailed account of SLPs attitude and practices in working with autistic children unique to multilingual Indian context. The findings suggest SLPs hold divergent attitudes toward bilingual intervention. SLPs reported use of limited strategies for addressing parental concerns. SLPs discussed factors for consideration of language choice, including the lack of culturally appropriate assessment tools. Our findings thus provide implications for SLPs, speech pathology training institutions, parents and policy makers to improve knowledge, training and practices for working with bi/multilingual children with ASD. These results extend findings on SLPs knowledge and practice from predominant western monolingual context to multilingual, multicultural Indian context and offer a starting point for future research in similar multilingual settings.

## Supporting information

S1 Appendix Interview guide used for the study. (DOCX)

S2 Appendix Initial codes and derived themes. (XLSX)
