# Supplementary material for: Speech language pathologists’ attitudes on bilingual practices for children with autism in India: A qualitative study
Source: PLoS One. 2026 Mar 26;21(3):e0343695. doi: 10.1371/journal.pone.0343695 (PMC13020778; doi:10.1371/journal.pone.0343695)
Supplement: S1 Appendix — (DOCX) [file pone.0343695.s001.docx]

**S1 Appendix**

**Interview Guide**

1. How often do you come across children with autism having bilingual exposure in your practice? What is your opinion about bilingualism?
2. How crucial is it for an SLP who is working with children on the autism spectrum to have bilingual exposure to know or speak their clients' native language? What options do you have when working with a client who doesn't speak your native language?
3. What is your opinion and experience of dual language exposure during language development in children with autism?
4. What suggestions would you provide to the parents of children with autism regarding language use? i.e., in favor of/against the choice of a bilingual approach, and why?
5. How can an SLP be better prepared for bilingual practice for children with autism?
